# Bearing Fault Diagnosis Method Based on RCMFDE-SPLR and Ocean Predator Algorithm Optimizing Support Vector Machine

**DOI:** 10.3390/e24111696

**Published:** 2022-11-20

**Authors:** Mingxiu Yi, Chengjiang Zhou, Limiao Yang, Jintao Yang, Tong Tang, Yunhua Jia, Xuyi Yuan

**Affiliations:** 1 School of Information Science and Technology, Yunnan Normal University, Kunming 650500, China; 2 The Laboratory of Pattern Recognition and Artificial Intelligence, Kunming 650500, China; 3 Faculty of Information Engineering and Automation, Kunming University of Science and Technology, Kunming 650500, China

**Keywords:** characteristic extraction, refine composite multiscale fluctuation dispersion entropy, self-paced learning and low-redundant regularization

## Abstract

For the problem that rolling bearing fault characteristics are difficult to extract accurately and the fault diagnosis accuracy is not high, an unsupervised characteristic selection method of refined composite multiscale fluctuation-based dispersion entropy (RCMFDE) combined with self-paced learning and low-redundant regularization (SPLR) is proposed, for which the fault diagnosis is carried out by support vector machine (SVM) optimized by the marine predator algorithm (MPA). First, we extract the entropy characteristics of the bearings under different fault states by RCMFDE and the introduction of the fine composite multiscale coarse-grained method and fluctuation strategy improves the stability and estimation accuracy of the bearing characteristics; then, a novel dimensionality-reduction method, SPLR, is used to select better entropy characteristics, and the local flow structure of the fault characteristics is preserved and the redundancy is constrained by two regularization terms; finally, using the MPA-optimized SVM classifier by combining Levy motion and Eddy motion strategies, the preferred RCMFDE is fed into the MPA–SVM model for fault diagnosis, for which the obtained bearing fault diagnosis accuracy is 97.67%. The results show that the RCMFDE can effectively improve the stability and accuracy of the bearing characteristics, the SPLR-based low-dimensional characteristics can suppress the redundancy characteristics and improve the effectiveness of the characteristics, and the MPA-based adaptive SVM model solves the parameter randomness problem and, therefore, the proposed method has outstanding superiority.

## 1. Introduction

As a key transmission component in rotating machinery, rolling bearings are widely used in important fields such as metallurgy, chemical industry, aerospace, and so on, and are also one of the main sources of failure of rotating machinery in large equipment such as gas turbines, air compressors, and wind power generation units. The operating environment of this equipment is mostly high temperature, high speed, heavy load, etc., which greatly improves the failure rate of rolling bearings, and its working state also has a direct impact on the safety of mechanical equipment operation. Cerrada M. et al. [1] pointed out that nearly 40% of mechanical equipment faults are caused by bearing faults; therefore, research on fault characteristic extraction and fault diagnosis methods of rolling bearings is particularly important. Effective fault characteristic extraction strategies and reliable fault diagnosis models can improve the safety and reliability of mechanical equipment and reduce maintenance and repair costs.

Traditional linear and stationary signal analysis methods inevitably have some limitations in the analysis of non-stationary and nonlinear signals [2]. Entropy is a measure that monitors kinetic mutations and time series randomness, which can not only measure the randomness between different variables, but also has the characteristics of good stability and strong noise immunity. Therefore, the entropy method is very suitable for characteristic extraction and characteristic analysis of nonlinear non-stationary signals. The existing entropy characteristic extraction methods include permutation entropy (PE) [3], approximate entropy (AE) [4], sample entropy (SM) [5], fuzzy entropy (FE) [6], multiscale entropy (MSE) [7], multiscale fuzzy entropy (MFE), etc. In 2016, Rostaghi et al. [8] proposed dispersion entropy (DE), which can overcome the defect of the original entropy value and detect the change in noise bandwidth, frequency, and amplitude. In 2018, Hamed et al. [9] further proposed multiscale dispersion entropy (MDE), which is a fast signal complexity analysis method. Zhang et al. [10] extracted the MDE characteristics of the bearing and applied them to the identification of the type and degree of fault. However, the coarse-grained process of MDE has the problem of information loss. In 2017, Hamed [9] proposed refined composite multiscale dispersion entropy (RCMDE), and the coarse-grained information loss problem of MDE was improved. Azami [11] verified that RCMDE has higher stability and reliability than MDE through Gaussian white noise and 1/f noise and logic diagrams. Refs. [12,13,14] applies RCMDE to bearing fault diagnosis and achieves good results. However, the dispersion entropy only considers the absoluteness of the amplitude without considering the relativity; therefore, it is unable to evaluate the fluctuation of the signal. Therefore, the accuracy and stability of the entropy estimation of bearing fault characteristics can still be further improved. In 2021, Hong Yang et al. [15] proposed refined composite multiscale fluctuation-based dispersion entropy (RCMFDE), and it was used for the characteristic extraction of ship radiation noise in complex marine environments. It can be seen from the analysis that RCMFDE has good characteristic extraction performance; however, there are still shortcomings such as characteristic redundancy and high dimension. In addition, RCMFDE only now, for the first time, being used for bearing fault characteristic extraction, and its use will provide a good method reference for fault diagnosis.

In this paper, RCMFDE is applied to the extraction of bearing fault characteristics; however, the extracted entropy characteristics have high dimension, characteristic redundancy, and characteristic noise. Therefore, characteristic dimension reduction and characteristic selection are particularly important. Manifold learning is a nonlinear dimensional reduction method that can more accurately reflect the nature of things. Commonly used manifold learning methods are isometric mapping (ISOMAP), local tangent space arrangement (LTSA), locally linear embedding (LLE), linear local tangent space arrangement (LLTSA), etc. Chen [16] applied the ISOMAP algorithm to the selection of eigenvalues. Lu et al. [17] applied LTSA for characteristic fusion to reduce redundant characteristics in high-dimensional characteristic sets. Zhang et al. [18] applied the improved LLE to the fault diagnosis of bearing data. Chen et al. [19] applied LLTSA to the study of planetary gear degradation state recognition. However, in the manifold learning method described above, part of the local structure information is easily lost during the characteristic extraction process, which ultimately affects the expression ability of the extracted main manifold structure. Since the original local neighborhood graph is destroyed, the neighborhood matrix must be reconstructed, which increases the number of calculations and leads to a long calculation time [20]. In 2022, Li et al. [21] proposed self-paced learning and low-redundant regularization (SPLR), which effectively solved the problems of the above method. SPLR utilizes a self-paced learning framework to exclude outliers, while introducing regularization terms in the subspace space learning framework to select low-redundancy characteristics. SPLR considers both characteristic and data diversity, and preserves the local manifold structure of the data. Therefore, in this paper, SPLR is used to extract superior RCMFDE low-dimensional characteristics, and the redundancy of the entropy characteristics is reduced and the reliability is improved.

After obtaining the fault entropy characteristic, it is also necessary to establish the correspondence between the entropy characteristic and the fault category through the classifier. Supervised learning refers to the machine learning method of learning predictive models from labeled data, in which classified learning is the most common supervised learning problem. Supervised learning is widely used in fault diagnosis, showing great advantages for marked sample data [22,23]. In common supervised learning methods, artificial neural networks can easily fall into local extreme value, and the speed of convergence is slow. The decision tree is prone to overfitting, ignoring correlations between attributes. Support vector machine (SVM) is a particularly powerful and flexible supervised learning model that analyzes data for both classification and regression [24], which has unique advantages in solving nonlinear and small-sample characteristic data. In addition, the SVM algorithm has good robustness and generalization, effectively avoiding local extreme value. Therefore, SVM has been widely used in fault diagnosis research, and SVM has also made remarkable achievements in other pattern recognition applications. Bie [25] uses resonance sparse signal decomposition and SVM to accurately identify the faults of gearboxes. Wang et al. [26] utilized integrated fault characteristics and support vector machines to provide both high-precision and fast fault detection. However, SVM also has some defects, such as that the diagnostic accuracy of SVM depends on the selection of kernel function and penalty parameters. Therefore, the optimization of SVM parameters is particularly important. Common parameter optimization algorithms include the genetic algorithm and particle swarm optimization algorithm. Genetic algorithm programming is complex, the search speed is slow, and the particle swarm algorithm will appear premature and easily fall into the local optimum. Compared with the traditional SVM optimization algorithm, in 2020, Faramarzi et al. [27] proposed the MPA algorithm to simulate the behavior between marine predators and prey. At present, MPA has been widely used in reservoir operations [28], frequency regulation in multi-microgrid systems [29], multilevel threshold image segmentation [30], and other fields. The MPA algorithm has faster convergence speed, stronger global search ability, and higher stability. Therefore, in this paper, MPA–SVM is used to establish the corresponding relationship between entropy characteristics and fault categories, and the accuracy of fault diagnosis has been greatly improved.

In summary, in order to solve the problems of bearing fault characteristics being difficult to accurately extract, high characteristic dimension, and low fault diagnosis accuracy, we propose a fault diagnosis method based on the RCMFDE-SPLR entropy characteristic set and MPA–SVM. Firstly, the entropy characteristics of bearings under different fault states are extracted by RCMFDE; then, a better entropy characteristic is selected by SPLR; finally, the fault characteristic set is fed into the MPA-optimized SVM model. The proposed method is applied to the experimental data analysis process of rolling bearing fault diagnosis, and the results show that the method can effectively and accurately distinguish the types of various working conditions. The innovations and contributions of this paper are summarized as follows:

(1) Aiming at the problem of information loss in the MDE coarse-grained process, a fine composite strategy is introduced to improve the stability of entropy characteristics. Aiming at the problem that the characteristic trend of RCMDE is ignored, the fluctuation distribution mode is introduced, and the accuracy of the entropy value is further improved;

(2) Aiming at the problem that the entropy characteristic dimension is too high and there is characteristic redundancy, we select effective low-dimensional characteristics through the latest and most effective SPLR. Those low-dimensional characteristics that are beneficial for identification are effectively retained, the burden of SVM fault diagnosis model is further reduced, and the computational complexity of the method is improved;

(3) A fault diagnosis method based on RCMFDE characteristic extraction, SPLR characteristic selection, and MPA–SVM is proposed, which improves the accuracy and effectiveness of mechanical equipment fault diagnosis.

The rest of this article is arranged as follows: in Section 1, the theory of refine composite multiscale fluctuations dispersion entropy is introduced; in Section 2, the theory of self-paced learning and low-redundant regularization is introduced; in Section 3, the marine predator algorithm and support vector machine theory are introduced; in Section 4, we describe the advantages of RCMFDE-SPLR characteristic optimization and the MPA–SVM fault diagnosis method in detail.

## 2. Refined Composite Multiscale Fluctuation-Based Dispersion entropy

### 2.1. Dispersion Entropy

Dispersion entropy simultaneously detects changes in bandwidth, frequency, and amplitude. The calculation time is also greatly shortened, and the anti-interference is strong, which can effectively reflect the fault characteristics of the bearing. The univariate time series with length N is expressed as:

(1) $$x=\left\{\left.x_{j}\right|j=1,2,\ldots ,N\right\}$$

(1) Map *x* to $y=\left\{\left.y_{j}\right|j=1,2,\ldots ,N\right\}y_{j}\in (0,1)$ using the normal distribution function.

(2) $$y_{j}=\frac{1}{\sigma \sqrt{2\pi }}\int_{-\infty }^{x_{j}}e^{\frac{-{\left(t-u\right)}^{2}}{2\sigma^{2}}}dt$$

where u is the expectation; σ is the standard deviation.

(2) Map each $y_{j}$ to an integer in the range of [1,c] using a linear algorithm:

(3) $$z_{j}^{c}=round\left(cy_{j}+0.5\right)$$

where round() is the rounding function; $z_{j}^{c}$ is the *j*-th element of the classification sequence $z^{c}$.

(3) Use formula (4) to calculate the embedding vector.

(4) $$z_{i}^{m,c}=\left[z_{i}^{c},z_{i+d}^{c},\ldots ,z_{i+(m-1)d}^{c}\right]i=1,2,\ldots ,N-(m-1)d$$

(5) $$z_{i}^{c}=v_{0},z_{i+d}^{c}=v_{1},z_{i+(m-1)d}^{c}=v_{m-1}$$

where m is the embedded dimension, d is the delay, and the total number of dispersion modes of $z_{i}^{m,c}$ is $c^{m}$.

(4) The probability of each dispersion mode $r_{v_{0}v_{1}\ldots v_{m-1}}$ is:

(6) $$p\left(r_{v_{0}v_{1}\ldots v_{m-1}}\right)=\frac{N(r_{v_{0}v_{1}\ldots v_{m-1}})}{N-(m-1)d}$$

(5) According to Shannon’s definition of information entropy, the dispersion entropy $E_{D}(x,m,c,d)$ can be expressed as:

(7) $$E_{D}(x,m,c,d)=-\sum_{r=1}^{c^{m}}p(r_{v_{0}v_{1}\ldots v_{m-1}})\ln p(r_{v_{0}v_{1}\ldots v_{m-1}})$$

### 2.2. Refined Composite Multiscale Fluctuation-Based Dispersion Entropy

Fluctuation dispersion entropy (FDE) is a normal cumulative distribution function (NCDF) map that calculates only the first-time scale. Multiscale fluctuation dispersion entropy (MFDE) is mainly based on FDE for the multiscale research of time series. However, the MFDE does not take into account that there is a certain relationship between the segmented data, and it is likely that the statistics will be lost and that there will be some deviation from the initial position. Compared with MFDE, the preprocessing process of raw data is further refined in RCMFDE. When the scale factor is τ, the original data are divided into several non-overlapping segments of length τ and the average of these segments is taken as the RCMFDE characteristic. The specific steps of RCMFDE are as follows:

Step 1: The *k*-th coarse-grained time series $x_{k}^{\tau }=\{x_{k,1}^{\tau },x_{k,2}^{\tau },\ldots ,x_{k,j}^{\tau }\}$ is calculated as follows:

(8) $$x_{k,j}^{\tau }=\frac{1}{\tau }\sum_{b=k+\tau (j-1)}^{k+j\tau -1}u_{b},1\leq j\leq \frac{N}{\tau },1\leq k\leq \tau$$

Step 2: *RCMFDE* is defined as follows:

(9) $$RCMFDE(m,c,x,d,\tau )=-\sum_{q=1}^{{\left(2c-1\right)}^{m-1}}p(q_{v_{0}v_{1}\ldots v_{m-1}})\ln\bar{p}(q_{v_{0}v_{1}\ldots v_{m-1}})$$

**Figure 1:**
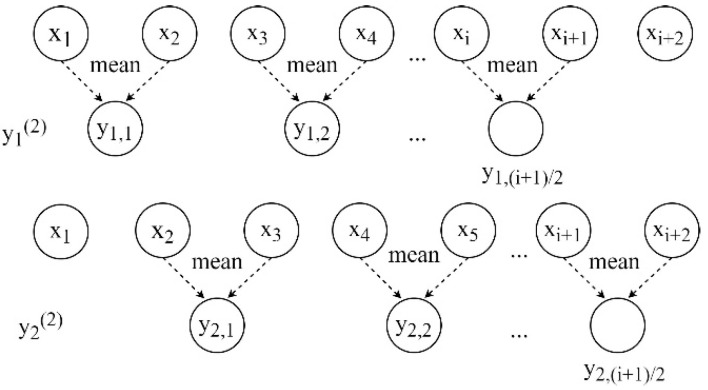
Coarse-grained process of *RCMFDE* (τ=2).

$\bar{p}\left(r_{v_{0}v_{1}\ldots v_{m-1}}\right)$ is the average value of the dispersion mode probability corresponding to the coarse-grained sequence, and the expression is as follows:

(10) $$\bar{p}\left(r_{v_{0}v_{1}\ldots v_{m-1}}\right)=\frac{1}{\tau }\sum_{k=1}^{\tau }p_{k}^{\left(\tau \right)}$$

where $p_{k}^{\left(\tau \right)}$ is the probability of the dispersion pattern corresponding to the *k*-th coarse-grained sequence at scale τ. The RCMFDE multiscale coarse-grained process is shown in Figure 1, where τ is set to 2.

*RCMFDE* averages multiple initial point positions, which can reduce the influence of initial point positions on entropy and avoid the loss of statistical information.

## 3. Self-Paced Learning and Low-Redundant Regularization

SPLR integrates a self-paced learning and subspace learning framework, and the redundancy of characteristics is constrained by two regularization terms and the local popular characteristics are retained. The SPLR algorithm parameters are shown in Table 1.

### 3.1. Self-Paced Learning Framework

In 2009, Bengio et al. [31] proposed curriculum learning. Based on this, M. Kumar et al. proposed self-paced learning (SPL) [32], for which the expression is as follows:

(11) $$\underset{w,v}{\min}E(w,v;\lambda )=\left(v_{i}L(y_{i},g(x_{i},w))\right)+f(v_{i},\lambda )$$

where $v_{i}$ is the weight of $x_{i}$, $f(v_{i},\lambda )$ is a regularization term, and $L(y_{i},g(x_{i},w)$ is the loss function, which represents the residual between the true label $y_{i}$ and the predicted $g(x_{i},w)$.

SPLR integrates hybrid regularizers in a self-paced learning framework. Not only can the importance of samples be distinguished but, also, when the losses of samples $x_{i}$ and $x_{j}$ are small enough, their weights are set to 1, which tolerates small errors. The expression of the hybrid regularizer and the corresponding solution are as follows:

(12) $$\begin{array}{l} f^{M}(v;\lambda ,\gamma )=\frac{\gamma^{2}}{v+\gamma /\lambda },\\ v*(\lambda ,\gamma ;l)=\left\{\begin{array}{l} 1,\;\;if\;\;l\leq {\left(\frac{\lambda \gamma }{\lambda +\gamma }\right)}^{2};\\ 0,\;\;if\;\;l\geq \lambda^{2};\\ \gamma (\frac{1}{\sqrt{l}}-\frac{1}{\lambda }),otherwise \end{array}\right. \end{array}$$

### 3.2. Low-Redundant Regularization

The learning framework of the low-redundancy subspace is as follows:

(13) $$\begin{array}{l} \arg\underset{W,H}{\min}\frac{1}{2}{\left\|X-XHW\right\|}_{F}^{2}+\lambda_{1}tr(S^{T}W1W^{T})\\ s.t.W\geq 0,H\geq 0,W^{T}W=E_{K} \end{array}$$

where the first term is the subspace learning framework, and the second term is the pairwise similarity regularizer. Based on the fact that similar samples existing in the original space should be similar when embedded in the subspace, the above objectives can be met by minimizing the following problems.

(14) $$\arg\underset{W}{\min}\frac{1}{2}\sum_{i,j}{\left\|x_{i}W-x_{j}W\right\|}_{2}^{2}z_{ij}=tr(W^{T}X^{T}LXW)$$

where, $z_{ij}$ is the similarity value between samples, L is the Laplace matrix, and D is the diagonal matrix. In combination with formulas (13) and (14), the expression of the low-redundancy subspace learning framework that preserves local manifold characteristics is as follows:

(15) $$\begin{array}{l} \arg\underset{W,H}{\min}\frac{1}{2}{\left\|X-XWH\right\|}_{F}^{2}+\lambda_{1}tr(S^{T}W1W^{T})+\lambda_{2}tr(W^{T}X^{T}LXW)\\ s.t.\;W\geq 0,H\geq 0,W^{T}W=E_{K} \end{array}$$

In addition, a sparsity regularization term is added, and by taking advantage of the advantages of $l_{2,1/2}$ norm, the final objective function of the proposed algorithm is expressed as follows:

(16) $$\begin{array}{l} \underset{W,H,v}{\min}\sum_{i=1}^{n}v_{i}{\left\|x_{i}=x_{i}WH\right\|}_{2}^{2}+\sum_{=1}^{n}\frac{\gamma^{2}}{v_{i}+\gamma /\eta }+\lambda_{1}tr(S^{T}W1W^{T})\\ +\lambda_{2}tr(W^{T}X^{T}LXW)+\alpha {\left\|W\right\|}_{2,1/2}^{1/2}\\ s.t.0\leq v_{i}\leq 1,i=1,2,\ldots ,n,W\geq 0,H\geq 0,W^{T}W=I_{K} \end{array}$$

where α>0, $\lambda_{1}>0$, and $\lambda_{2}>0$ are trade-off parameters and γ>0 is an interval control parameter. The first and second terms in formula (16) are intended to maintain the global reconstruction information and mitigate the influence of noise to some extent. The third item is introduced to reduce redundancy between characteristics. The fourth item is for the preservation of local geometry. The fifth term aims to promote row sparsity of the projection matrix.

## 4. Adaptive SVM Model Based on Ocean Predator Algorithm

### 4.1. Support Vector Machine Theory

A small sample classification method maximizes the distance between different classes of samples on both sides of the hyperplane by constructing an optimal hyperplane. When using support vector machines to make correlation predictions, for a given training set, we always hope to get a regression modelsuch as $f(x)=\omega^{T}\varphi (x)+b$, so that f(x) is as close as possible to the actual value y, the weight vector w and the offset b are the model parameters to be determined, and φ(x) is a nonlinear mapping. The dual problem of SVM can be obtained by introducing a Lagrange multiplier:

(17) $$\begin{array}{l} \max\sum_{i=1}^{m}({\hat{\alpha }}_{i}-\alpha_{i})y_{i}-\sum_{j=1}^{m}\left({\hat{\alpha }}_{j}+\alpha_{j}\right)\varepsilon \\ -\frac{1}{2}\sum_{i=1}^{m}\sum_{j=1}^{m}\left({\hat{\alpha }}_{i}-\alpha_{i}\right)({\hat{\alpha }}_{j}+\alpha_{j})K(x_{i},x_{j}) \end{array}$$

where $\alpha_{i}$, ${\hat{\alpha }}_{i}$, $\alpha_{j}$ and ${\hat{\alpha }}_{j}$ are Lagrange multipliers; $K(x_{i},x_{j})$ is the kernel function.

Solving formula (17) can obtain the SVM regression model:

(18) $$f(x,{\hat{\alpha }}_{i},\alpha_{i})=\sum_{i=1}^{m}({\hat{\alpha }}_{i}-\alpha_{i})K(x_{i},x_{j})+\hat{b}$$

### 4.2. Marine Predator Optimization Support Vector Machine

MPA is mainly inspired by the foraging strategies of marine predators—the optimal encounter strategies of Levy and Eddy motions and interactions between predator and prey. Levi’s flight includes random motions of small-step walks and occasional large-step long jumps, which can be accurately and deeply searched. Brownian motion can track and explore distant regions. MPA initializes the prey location by searching the spatial range:

(19) $$X_{0}=X_{\min}+rand(X_{\max}-X_{\min})$$

where, $X_{\max}$ and $X_{\min}$ are the upper and lower limits of the search space, respectively; rand( ) is a random number in the range of [0,1].

**Figure 2:**
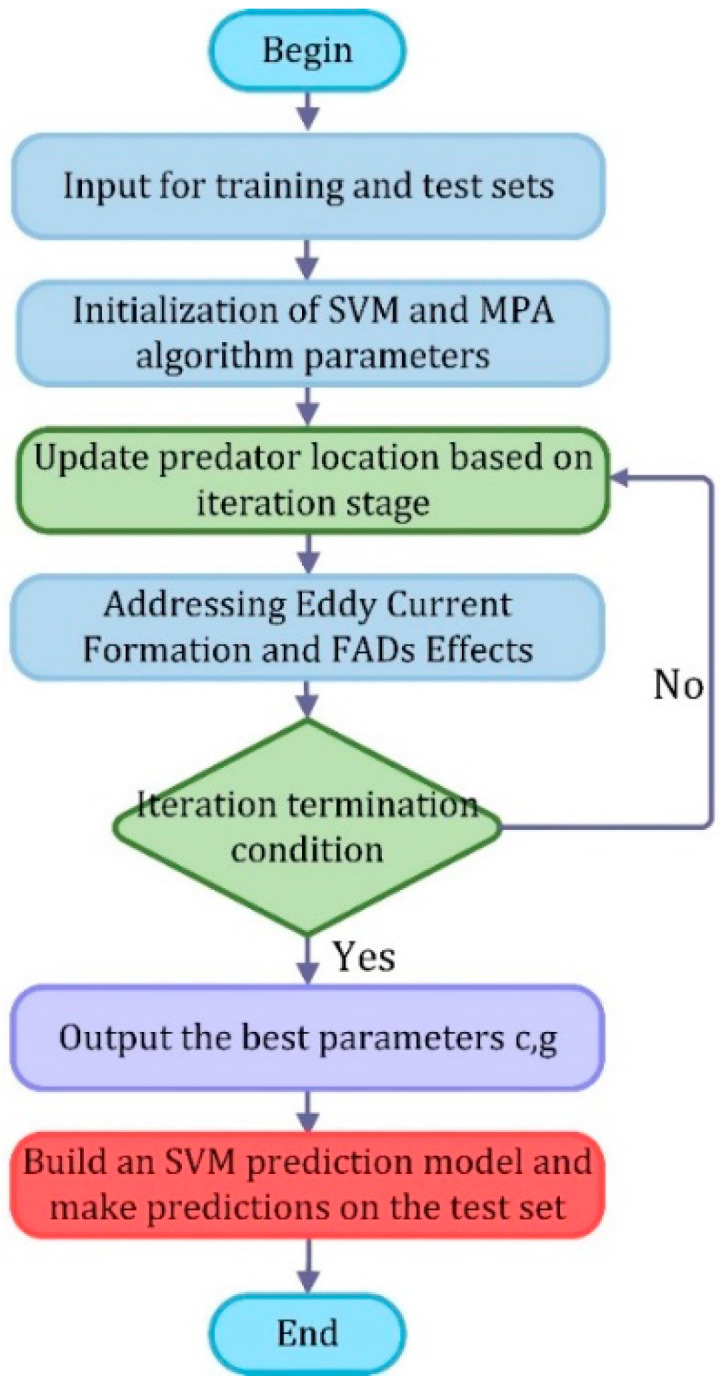
MPA–SVM process.

In view of the advantages of marine predator optimization algorithm, which is applied to the optimization process of SVM, and the flow chart of marine predator optimization support vector machine is shown in Figure 2. The specific steps are described as follows:

**Step 1:** Input the samples to the SVM;

**Step 2:** Set the algorithm parameters and initialize the population;

**Step 3:** Calculate the fitness value of prey matrix P, record the optimal position, and calculate the predator matrix E;

**Step 4:** According to the iteration stage, the predator selects the corresponding update method to update the predator position;

**Step 5:** Calculate the fitness value and update the optimal position;

**Step 6:** To solve the eddy current formation and FADs effects, the algorithm jumps out of the local optimal solution as much as possible during the iteration process;

**Step 7:** Determine whether the stopping condition is satisfied, if not, repeat steps 3~6, otherwise output the optimal result of the algorithm (c, g);

**Step 8:** Build a predictive model of the SVM and make predictions about the test set.

## 5. Experimental Analysis

### 5.1. Experimental Data and the Method of This Paper

**Figure 3:**
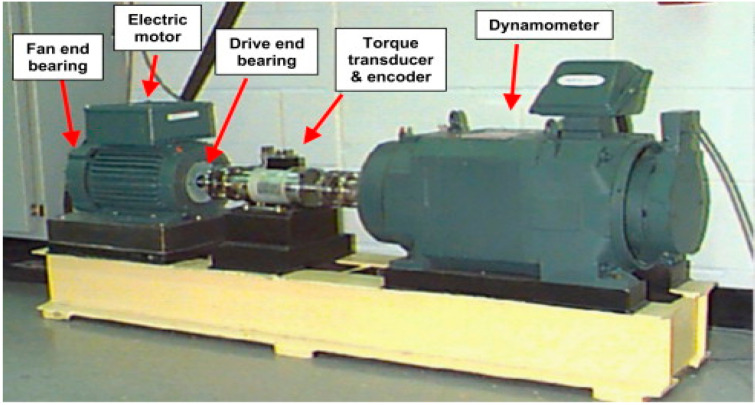
Case Western Reserve University rolling bearing testing platform and schematic diagram.

In order to get closer to the slight stage of rolling bearing fault, the bearing data of Case Western Reserve University in the United States were used to verify the effectiveness and accuracy of the diagnosis method proposed in this paper. Figure 3 shows the rolling bearing experimental platform of Case Western Reserve University. In order to enhance the persuasiveness, for the selection of vibration signal, the influence of bearing fault type and fault degree were fully considered in the experiment. The sampling frequency of the data selected in the experiment was fs = 12,000 Hz, the bearing test speed was 1797 r/min, and the motor load (horsepower) was 0. These data are vibration data collected by the accelerometer. The accelerometer was placed on the drive end, fan end and base of the motor housing; therefore three different types of data were generated. The acceleration data of the driving end was selected for the experiment.

**Figure 4:**
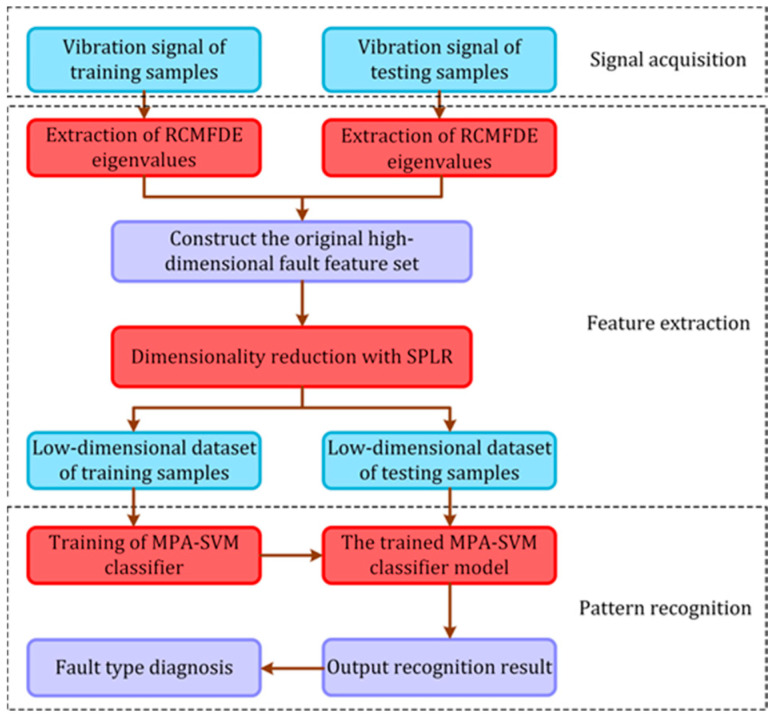
Flow chart of fault diagnosis method.

The experimental data specifically include vibration signals under four different working conditions (normal state, inner ring fault, outer ring fault, and rolling element fault). Table 2 details the label information of the experimental data. Among them, each group of fault data has 120,000 sampling points. Each sample takes 2000 sampling points, and each group of fault data has 60 samples. There are 10 groups of fault data with a total of 600 samples. A total of 1~60 are normal data; 61~120, 121~180, and 181~240 are inner ring fault data; 241~300, 301~360, and 361~420 are outer ring fault data; 421~480, 481~540, and 541~600 are the rolling element fault data. In this paper, a fault diagnosis method for RCMFDE-SPLR entropy characteristic optimization and MPA–SVM classifier is established. The process is shown in Figure 4. Specific steps are as follows:

**Step 1*:*** Under the condition that the sampling frequency *f_s_* is 12 k, 60 groups of vibration acceleration signals of 10 different working conditions are collected; 30 groups are selected as training samples under each working state, and the remaining 30 groups are used as test samples;

**Step 2:** Calculate the RCMFDE entropy values of the training and test samples through the RCMFDE algorithm, and combine them into the original high-dimensional fault characteristic set $F^{\left(10\times 60\right)\times \tau }$, where τ is the scale factor;

**Step 3:** Compress the original high-dimensional characteristic set through the SPLR algorithm to obtain a low-dimensional sensitive characteristic vector $F\prime^{\left(10\times 60\right)\times \tau }$ where *d* is the intrinsic dimension;

**Step 4**: The low-dimensional characteristic sets of training samples and test samples are input into the MPA–SVM multi-fault classifier to identify and diagnose the type of working condition.

### 5.2. Experiment Analysis of RCMFDE Characteristic Extraction

**Figure 5:**
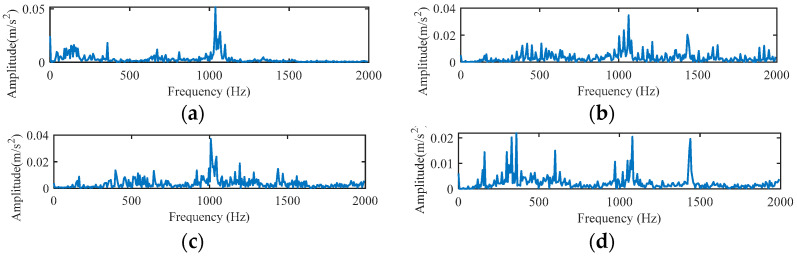
Raw vibration signal spectrum. (**a**) NOR, (**b**) IRF, (**c**) ORF, (**d**) BF.

**Figure 6:**
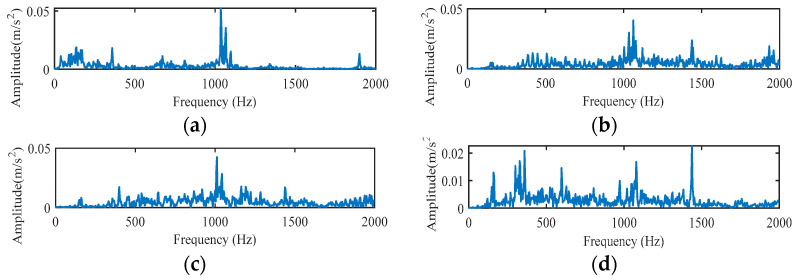
RCMFDE coarse-grained sequence spectrum. (**a**) NOR, (**b**) IRF, (**c**) ORF, (**d**) BF.

In a complex environment, the key of characteristic extraction is to obtain more representative target characteristic parameters. When the rolling bearing of the motor breaks down, the amplitude of the vibration signal will change correspondingly, and the entropy value can accurately identify this change. Therefore, it is necessary to introduce the concept of entropy. First, the vibration signal is multi-scaled to obtain the RCMFDE coarse-grained time series. The experiment intercepts the vibration signal of the bearing in normal state with a data length of 2000 and the vibration signal of the inner ring fault, outer ring fault, and rolling element fault with a fault size of 0.007 mm, and draws the original vibration signal spectrum and the RCMFDE coarse-grained sequence spectrum. Figure 5 is the spectrum of the original vibration signal, and Figure 6 is the spectrum of the RCMFDE coarse-grained sequence. In the figure, NOR, IRF, ORF, and BF represent normal signal, inner ring fault, outer ring fault, and rolling element fault, respectively. Among them, the scale factor of RCMFDE is τ=3, the sampling frequency is $f=f_{s}/3$, that is, *f* = 4000 Hz, and the spectral bandwidth is 2000 Hz. It can be seen from the figure that the RCMFDE coarse-grained sequence spectrum is basically the same as the original vibration signal spectrum.

**Figure 7:**
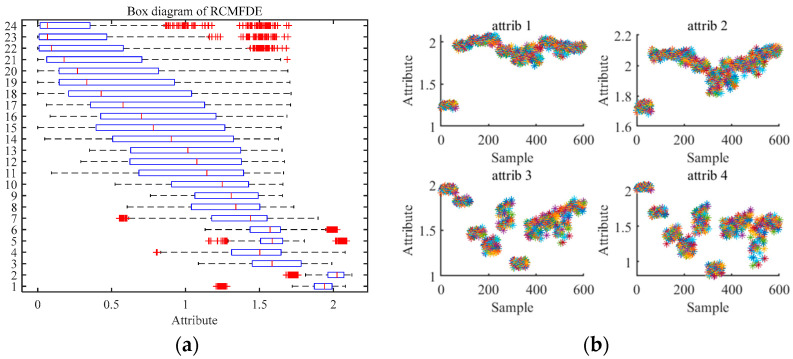
RCMFDE analysis results of different working conditions of rolling bearings. (**a**) Box diagram of RCMFDE; (**b**) Multidimensional visualization of RCMFDE.

**Figure 8:**
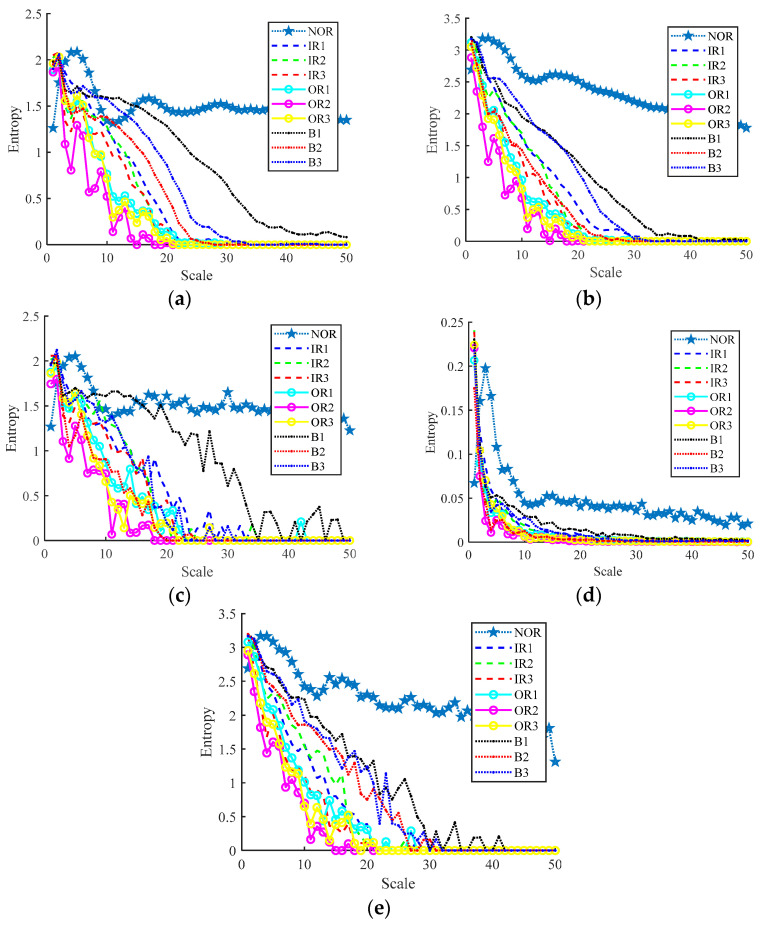
Analysis curve of entropy value under 10 different working states. (**a**) RCMFDE, (**b**) RCMDE, (**c**) MFDE, (**d**) MFE, (**e**) MDE.

**Figure 9:**
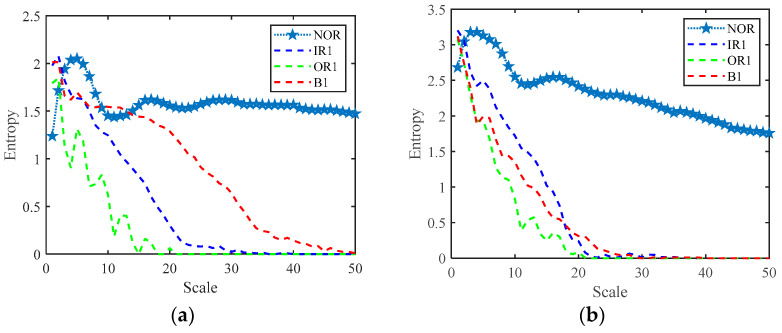

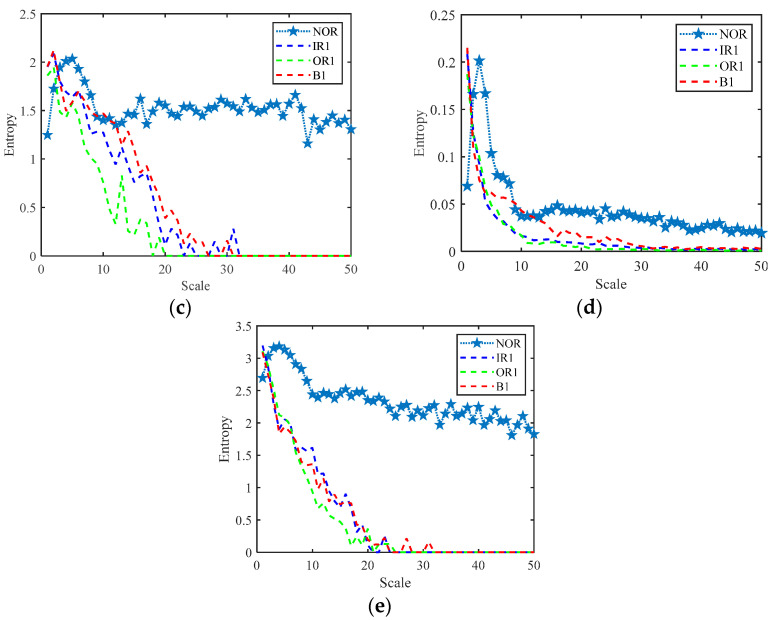
Entropy curve under different working conditions. (**a**) RCMFDE, (**b**) RCMDE, (**c**) MFDE, (**d**) MFE, (**e**) MDE.

Then, we extract the RCMFDE entropy characteristic. Taking the first sample, NOR, as an example, Table 3 shows the extracted first 24-dimensional entropy characteristics. Figure 7 shows the analysis results of 10 groups of RCMFDE entropy characteristics with different working states. Figure 7a is a box diagram of the 24-dimensional entropy of the first fault data, which shows the range of entropy. The graph reflects the central position and dispersion range of 24-dimensional entropy features. The red mark in the figure indicates the outlier of the dimensional feature, that is, the value separated from other entropy values. The outlier of the first dimension data in Figure 7a corresponds to the value of Abscissa 1~60 of attrib1 in Figure 7b. Figure 7b shows the visualization of the entropy values in the first four dimensions, and the entropy values of 10 different working states are shown in each dimension. Among them, the abscissa represents the sample, and the ordinate is the entropy value. Every 60 samples represent a running state. Furthermore, 1~60 represent normal samples, 61~240, 241~420, and 421~600 represent inner ring fault, outer ring fault, and rolling element fault samples, respectively. It can be seen from the figure that there is partial aggregation between the samples, and the normal samples are separated from the rest of the samples. In order to verify the characterization performance of RCMFDE for bearing fault characteristics, we also extracted the RCMDE, MFDE, MFE, and MDE fault characteristics of each signal sample for comparative analysis. Figure 8 is the entropy analysis curve of 10 groups of different working states. It can be roughly seen that the RCMFDE entropy curve is relatively sparse, which can better characterize the characteristics of different working states. In Figure 8a, when the scale factor is less than 1, the RCMFDE entropy values for normal working conditions are lower than the other entropy values. With the increase in scale, the entropy curves of different running states appear as obvious separation, and, for the same fault type, the trend of entropy curves is roughly the same. Figure 9 is the entropy curve of four different working conditions: normal, inner ring, outer ring, and rolling element. In Figure 9a, when the scale factor is greater than 1, the entropy curves of different working conditions have no cross phenomenon. In general, the RCMFDE entropy value of the normal state is larger than the restof the entropy values in most cases. The RCMFDE entropy curve is distinguishable.

**Figure 10:**
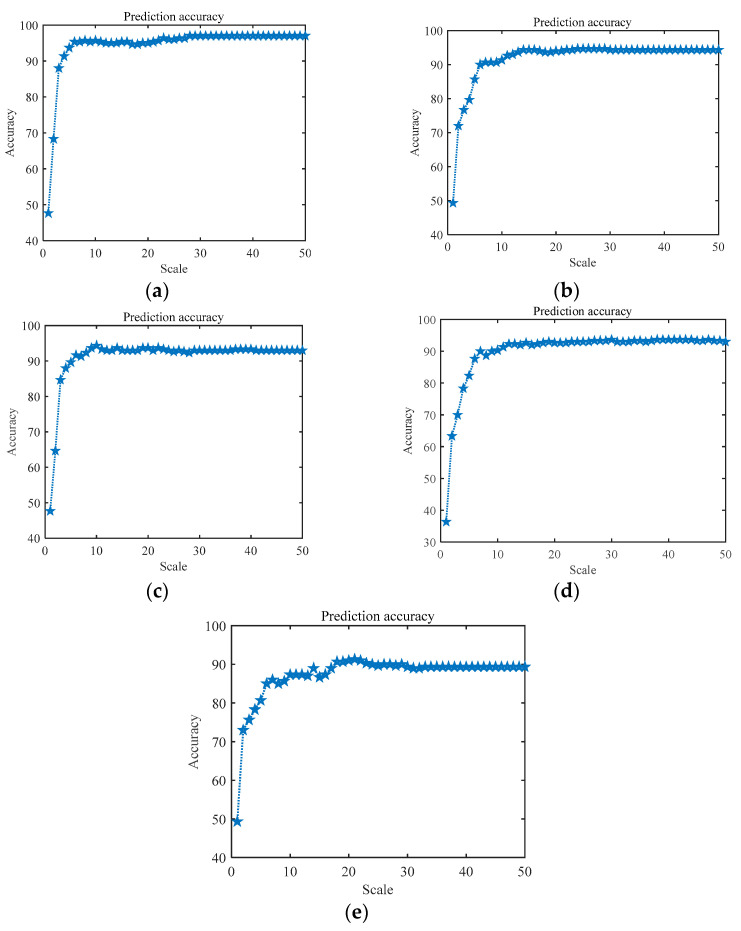
Recognition accuracy of multiple entropy values with different scale factors. (**a**) RCMFDE, (**b**) RCMDE, (**c**) MFDE, (**d**) MFE, (**e**) MDE.

**Figure 11:**
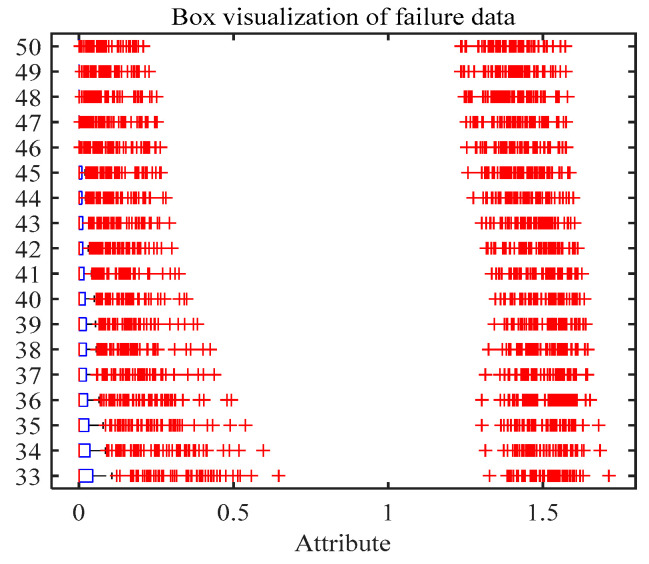
Visualization 32~50-dimensional characteristic box.

To illustrate the superiority of RCMFDE bearing characteristics, we input the extracted entropy characteristics into a classifier for experimental analysis. In the experiment, the embedding dimension m = 2, the delay tau = 1, and the number of categories c = 5. In order to verify the influence of the scale factor τ on the experimental results, we analyze the experimental results of different scale factors τ. Figure 10 shows the recognition accuracy of multiple sets of entropy characteristics (RCMFDE, RCMDE, MFDE, MDE, and MFE) at different τ. As can be seen from the figure, compared with other entropy characteristics, RCMFDE has the highest recognition accuracy. At the same time, it can be seen that, with the increase in the scale factor τ, that is, the increase in the characteristic dimension of entropy value, the accuracy of MPA–SVM recognition has fluctuated slightly. As shown in Figure 11 below, there are many outliers in the generated 32–50-dimensional characteristics, which means invalid characteristics. Therefore, the predicted recognition rate eventually reaches a stable value. It can be seen from Figure 10a that the recognition accuracy reaches 97% with the increase in the RCMFDE entropy dimension. RCMFDE improves the stability and noise resistance of entropy characteristics, and the accuracy of entropy values is further improved, making it very suitable for characteristic extraction of bearing vibration signals in complex industrial environments.

### 5.3. Experimental Analysis of SPLR Characteristic Optimization

The scale factor τ is set to 50 above. Therefore, a 50-dimensional entropy value characteristic is extracted. Although the RCMFDE entropy characteristic has achieved good results, the characteristic set still has redundant information and reduces the recognition accuracy. Therefore, this paper reduces the dimensionality of the RCMFDE fault characteristic set through self-rhythm learning and low-redundant regularization (SPLR). In other words, we select the optimal subset from the original characteristic space. SPLR is a dimensionality reduction method that considers the training order of samples. At the same time, SPLR eliminates the noise and redundancy characteristics that may suppress the performance in the training process. Specifically, combining self-paced learning and subspace learning frameworks not only reduces the effect of noise, but also makes the reconstructed information more accurate. The basic idea of SPLR is to rank entropy characteristics according to their importance. It can be seen from Figure 11 that the 33–50-dimensional characteristics are mostly outliers. This results in SPLR not being able to obtain a unique sequence of important characteristics. Therefore, the RCMFDE entropy value characteristics of from 1 to 32 dimensions are selected in the experiment. In the experiment, the algorithm runs for 1.873 s, which verifies the efficiency of this method. In the experiment, 30 sets of data under different working conditions are selected as the training set, and the other 30 sets of data are used as the test set. In order to evaluate the influence of SPLR on the fault recognition rate, we input the selected fault characteristics into MPA–SVM for fault diagnosis.

For SPLR, there are five parameters that need to be studied, namely α, $\lambda_{1}$, $\lambda_{2}$, $\lambda_{3}$, and γ. According to the literature [33], γ is fixed to 2. Weiyi Li et al. [20] did multiple sets of experiments, and the results showed that, with the change of α and $\lambda_{3}$, the clustering accuracy was relatively stable; however, with the change of $\lambda_{1}$ and $\lambda_{2}$, the clustering accuracy fluctuated to a certain extent. That is, SPLR is not sensitive to α and $\lambda_{3}$, but is sensitive to $\lambda_{1}$ and $\lambda_{2}$. A smaller $\lambda_{1}$ means that more redundant characteristics are selected, and a larger $\lambda_{1}$ means that the redundancy between characteristics is as small as possible but the regularization term is too strong. The smaller the $\lambda_{2}$ is, the more weakly the local geometric structure is preserved. The larger the $\lambda_{2}$ is, the more strongly the local geometric structure is preserved; however, the regularization term is too strong. Therefore, with the increase in the values of $\lambda_{1}$ and $\lambda_{2}$, no matter how many characteristics are selected, the corresponding clustering accuracy will first rise and then fall.

**Figure 12:**
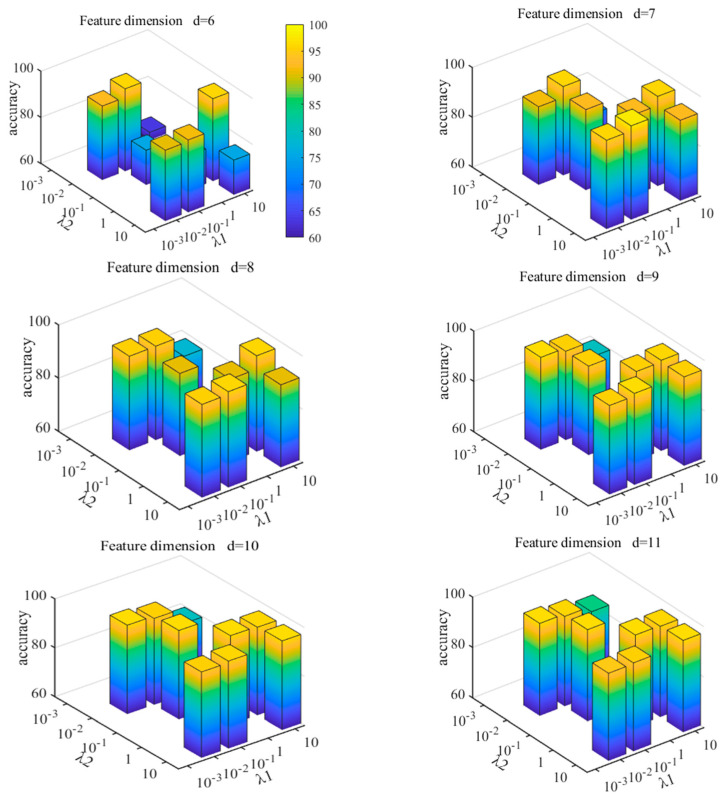
MPA−SVM recognition accuracy under different λ values.

In this experiment, the SPLR parameters are fixed as α=1, $\lambda_{3}=1$, the interval control parameter is β=2, the update parameter of k is μ=1.05, the maximum number of iterations is maxIter=50, the subspace dimension is K=600, and K is consistent with the number of samples. Considering that over-fitting will occur when $\lambda_{1}$ and $\lambda_{2}$ take {$10^{2}$, $10^{3}$}, this paper selects parameters from {$10^{-3}$, $10^{-2}$, $10^{-1}$, 1, $10^{1}$} and conducts experimental analysis. If the resulting dimensionality reduction sequence is not unique in the experiment, the accuracy is set to a null value. The results are shown in Figure 12, where τ represents the characteristic dimension after dimensionality reduction.

It can be seen from the figure that, when $\lambda_{1}{=10}^{-1}$ and $\lambda_{2}=10$, the experiment has achieved ideal results. Therefore, in this paper, $\lambda_{1}{=10}^{-1}$, $\lambda_{2}=10$. In order to explore the influence of the number of iterations on the experimental results, maxIter is set to 50, 100, 150, and 200, respectively, in the experiment, while other parameters are fixed. The results are shown in Table 4. It can be seen from the table that the number of iterations, 50, has achieved the best results in multiple characteristic dimensions. Among them, the accuracy of the characteristic prediction of the first seven dimensions reached 97.67%. This shows that SPLR can extract more favorable low-dimensional fault characteristics, and the burden of fault diagnosis model is further reduced. At the same time, when d=32, the prediction accuracy reaches 97%, which shows that the MPA–SVM algorithm can avoid the redundancy of entropy characteristics to a certain extent. Although more entropy characteristics can improve the diagnostic accuracy, the experimental results verify that SPLR can effectively reduce high-dimensional characteristic sets and improve the robustness of the learning method.

### 5.4. Experimental Analysis of Bearing Fault Diagnosis

**Figure 13:**
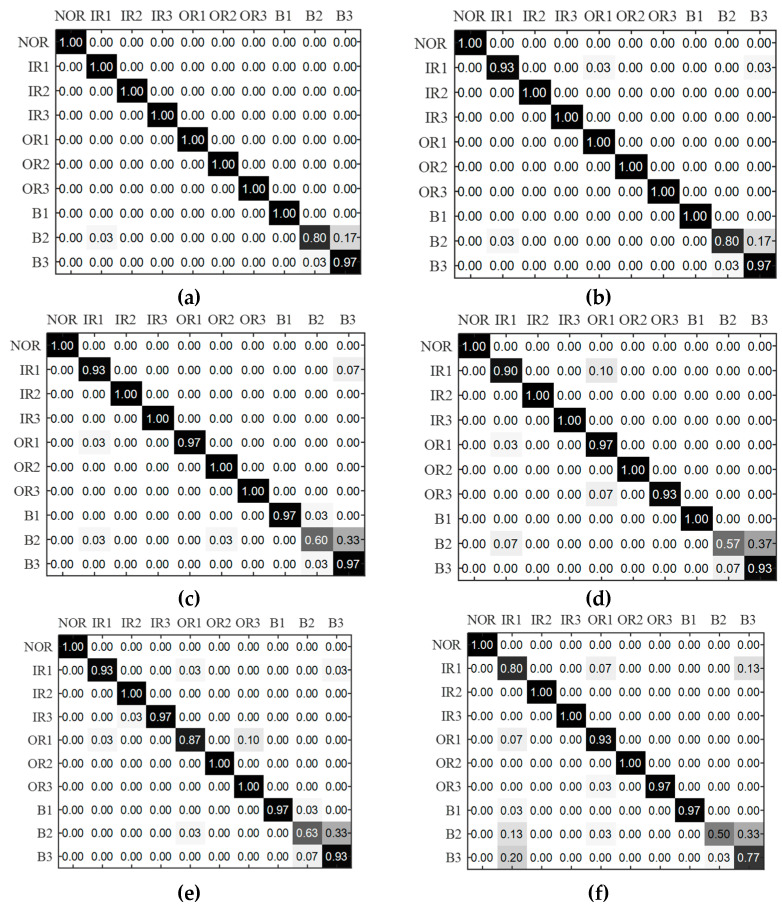
Recognition rate confusion matrix under MPA–SVM for each entropy value feature. (**a**) RCMFDE-SPLR, (**b**) RCMFDE, (**c**) RCMDE, (**d**) MFDE, (**e**) MFE (**f**) MDE.

We randomly select 50% of the RCMFDE-SPLR characteristic samples as the training set and 50% as the test set, that is, 300 training samples and 300 test samples. Subsequently, the bearing fault types are diagnosed by the MPA–SVM model. To verify the superiority of the RCMFDE-SPLR method, we compare it with RCMFDE, RCMDE, MFDE, MFE, and MDE entropy extraction methods. In the MPA–SVM fault diagnosis model, the penalty parameter is c=4.0007, and the kernel parameter is g=4.0006. The recognition rate of the fault classifier to the test samples is shown in Table 5. For the MPA–SVM multi-classification model, the proposed RCMFDE-SPLR characteristic obtains the highest diagnostic accuracy. In addition, the accuracy of RCMFDE is higher than that of MFDE, and the accuracy of RCMDE is higher than that of MDE. The results show that the fine composite coarse-grained strategy reduces the coarse-grained loss of vibration signals and improves the entropy stability. The accuracy obtained by RCMFDE is higher than that obtained by MFDE, and the accuracy obtained by MFDE is higher than that obtained by MDE, indicating that the introduction of the fluctuation strategy can preserve the amplitude information of the vibration signal sequence, which is more conducive to fault diagnosis. The accuracy obtained by the four characteristics of MDE, MFDE, RCMDE, and RCMFDE in Table 5 is 89.33%, 93%, 94.33%, and 97%, respectively. Figure 13 is the multi-category confusion matrix obtained by MPA–SVM. If the MDE characteristic is extracted, there will be misdiagnosis among the six states of IR1, OR1, OR3, B1, B2, and B3, and the fault mode is difficult to accurately identify. If the characteristics of MFDE and RCMDE are extracted, there will be misdiagnosis among the five states. Compared with MFDE and RCMDE characteristics, if RCMFDE characteristics are extracted, the probability of B2 being misdiagnosed as B3 is reduced by 20% and 16%, respectively. If RCMFDE-SPLR characteristics are extracted, the problem that 7% of IR1 samples in RCMFDE characteristics are misdiagnosed as OR1 and B3 states is resolved. The experimental results show that the fine composite coarse-grained strategy and the fluctuation dispersion entropy can effectively characterize the bearing fault characteristics. At the same time, the RCMFDE-SPLR method can effectively improve the accuracy of bearing fault diagnosis.

In order to verify the superiority of the MPA–SVM classifier over the fault diagnosis of the motor rolling bearing, we input the extracted entropy characteristics into the SVM and MPA–SVM classifier for diagnostic identification. Under the same entropy characteristics, the diagnostic accuracy of the MPA–SVM model is greater than that of the SVM model. Compared with the fault diagnosis method of MDE combined with SVM, the fault diagnosis method of RCMFDE-SPLR combined with MPA–SVM improves the diagnosis accuracy from 87.33% to 97.67%, which is improved by 10.34%. Moreover, we selected a sampling frequency of fs = 12,000 Hz, bearing test speed of 1772 r/min and 1730 r/min, and corresponding motor load (horsepower) of 1 and 3, respectively, for the experiment. The recognition rate of the fault classifier to the test sample is shown in Table S1. Among them, Figures S1 and S2 are their corresponding confusion moment matrices. The results show that the RCMFDE-SPLR characteristic extraction method and the MPA–SVM fault identification mode proposed in this paper are very effective.

In general, we can draw the following three conclusions: (1) The recognition rate size relationship of the five entropy characteristic extraction methods is: RCMFDE > MFDE > RCMDE > MDE > MFE; (2) compared with the RCMFDE characteristic extraction method (the recognition rate is 97%), the RCMFDE-SPLR characteristic extraction method can improve the recognition accuracy (increased by 1.33% and 0.67%, respectively) under both SVM and MPA–SVM fault classifiers; (3) the fault recognition rate of the MPA–SVM multi-fault classifier for the six entropy characteristics is higher than those of RCMFDE + SVM, RCMDE + SVM, MFDE + SVM, MFE + SVM, and MDE + SVM.

There are three reasons for the above conclusion: (1) RCMFDE not only considers the amplitude information of the vibration signal, but also overcomes the defect that the entropy error will increase with the increase in the scale factor, so it has high diagnostic accuracy; (2) the RCMFDE-SPLR characteristic extraction method removes the redundant information of the characteristic; therefore, the recognition effect is better than the original RCMFDE entropy characteristic, and the superiority of the SPLR algorithm is also verified. (3) The MPA algorithm combines Levy flight and Brownian motion, and adopts different random walk methods in different predation stages to achieve a balance between exploration and development, which improves the SVM fault identification rate.

## 6. Conclusions

In complex operating environments such as high temperature and high speed, bearing parts are damaged by coupling phenomenon and are interfered by noise, resulting in nonlinear and non-stationary signals. In a complex operating environment such as those having high temperature and high speed, the coupling phenomenon of bearing parts occurs and is interfered with by noise, resulting in nonlinear and non-stationary signals. However, the traditional MDE fault characteristic has three defects, that is, it does not consider the relationship between segmented data, the loss of amplitude information, or the poor stability of noise resistance. In addition, the SVM model is greatly affected by parameters and the recognition accuracy is reduced. Therefore, this paper proposes the fault characteristic extraction method of RCMFDE-SPLR, and constructs the MPA–SVM diagnosis model. The diagnostic results of the bearing verify the superiority of the proposed method, and the main work and analysis conclusions of this paper are as follows:

(1) In the bearing diagnosis experiment, the accuracy of the method based on RCMDE and SVM is 89%, which is higher than that of the method based on MDE and SVM, which is 87.33%. The experiments show that the composite coarse granularity suppresses the loss of information and improves the noise resistance of the fault features of mechanical parts. In addition, after introducing the fluctuation strategy, the accuracy of RCMFDE combined with SVM is improved to 94%. RCMFDE improves the stability of features, which further improves the fault diagnosis accuracy. The proposed RCMFDE solves the problems of loss of MDE feature information and the poor stability of noise resistance, which can effectively identify and diagnose bearing faults, providing an idea for on-line monitoring of motor rolling bearing faults under the background of strong noise.

(2) In the bearing diagnosis experiment, SPLR is added to select effective low-dimensional characteristics from the extracted entropy characteristics. The method of RCMFDE-SPLR combined with SVM improves the accuracy to 95.33%. It is shown that the subspace learning and the low-redundant regularization framework suppress the redundancy of entropy characteristics, and the favorable characteristics are retained, which further improves the diagnostic recognition accuracy.

(3) In bearing fault diagnosis, the method of RCMFDE-SPLR combined with MPA–SVM improves the diagnosis accuracy to 97.67%. In summary, the accuracy of bearing fault diagnosis has been increased from 89% to 97.67%, an increase of 8.67%. In addition, in the case of the same entropy value characteristics, the diagnostic accuracy of the LPV-SVM model based on Levy and Eddy is better than that of the SVM fault identification model, and the classification accuracy and efficiency are improved. It is shown that the proposed method can effectively diagnose different fault types of rolling bearings in 10 fault types.

## Figures and Tables

**Table 1:** SPLR algorithm parameters.

Notation	Description
$X\in R^{n\times d}$	Original data matrix
$W\in R^{d\times K}$	Projection matrix
$H\in R^{K\times d}$	Reconstruction matrix
$S\in R^{n\times n}$	Similarity matrix of characteristic space
$Z\in R^{d\times d}$	Similarity matrix of data space
tr(X)	The trace of *X*
${\left\|X\right\|}_{F}$	$\mathrm{The}\;\mathrm{Frobenius}\;\mathrm{norm}\;\mathrm{of}\;X,\;i.e.,\;{\left\|X\right\|}_{F}=\sqrt{\sum_{i=1}^{n}\sum_{j=1}^{d}x_{ij}^{2}}$
${\left\|X\right\|}_{p,q}$	$\mathrm{The}\;\mathrm{Frobenius}\;\mathrm{norm}\;\mathrm{of}\;X,\;i.e.,\;{\left\|X\right\|}_{p,q}={\left(\sum_{i=1}^{n}{\left(\sum_{j=1}^{d}{\left|x_{ij}\right|}^{p}\right)}^{\frac{q}{p}}\right)}^{\frac{1}{q}}$

**Table 2:** Bearing experimental data.

Category	Size/mm	Abbreviation	Rotating Speed/(r/min)	Label
Normal	0	NOR	1797	1
Inner ring failure	0.007	IR1	1797	2
	0.014	IR2	1797	3
	0.021	IR3	1797	4
Outer ring failure	0.007	OR1	1797	5
	0.014	OR2	1797	6
	0.021	OR3	1797	7
Balling failure	0.007	B1	1797	8
	0.014	B2	1797	9
	0.021	B3	1797	10

**Table 3:** RCMFDE values for different dimensions.

Number	1	2	3	4	5	6	7	8
RCMFDE	1.2393	1.7110	1.9494	2.0398	2.0500	1.9940	1.8413	1.6508
Number	9	10	11	12	13	14	15	16
RCMFDE	1.4647	1.3704	1.3503	1.3645	1.4019	1.4648	1.5402	1.6051
Number	17	18	19	20	21	22	23	24
RCMFDE	1.6361	1.6307	1.6174	1.6081	1.5910	1.5856	1.5920	1.6009

**Table 4:** MPA–SVM recognition accuracy under different iterations.

Accuracy	*d* = 7	*d* = 8	*d* = 9	*d* = 10	*d* = 32
maxIter = 50	97.67%	96%	96.33%	95.67%	97%
maxIter = 100	95.67%	96.67%	95.33%	95%	97%
maxIter = 150	96.67%	96.67%	95.33%	95.33%	97%
maxIter = 200	95%	95.33%	95.33%	94.67%	97%

**Table 5:** Fault recognition rate of each information entropy method.

Method	SVM Recognition Rate	MPA–SVM Recognition Rate
**MDE**	87.33%	89.33%
**MFE**	91%	93%
**MFDE**	93%	93%
**RCMDE**	89%	94.33%
**RCMFDE**	94%	97%
**RCMFDE-SPLR**	95.33%	**97.67%**

## Data Availability

The data used to support the findings of this study are available from the corresponding author upon request.

## Supplementary Materials

The following supporting information can be downloaded at: https://www.mdpi.com/article/10.3390/e24111696/s1, Table S1: Analysis of fault recognition rate results under MPA-SVM for RCMFDE-SPLR of different data; Figure S1: Recognition rate confusion matrix under MPA-SVM for RCMFDE-SPLR of data 1; Figure S2: Recognition rate confusion matrix under MPA-SVM for RCMFDE-SPLR of data 2.
