# Supplementary material for: Bearing Fault Diagnosis Method Based on RCMFDE-SPLR and Ocean Predator Algorithm Optimizing Support Vector Machine
Source: Entropy (Basel). 2022 Nov 20;24(11):1696. doi: 10.3390/e24111696 (PMC9688966; doi:10.3390/e24111696)
Supplement: Supplementary file 1 [file entropy-24-01696-s001.zip › entropy-1976826-supplementary.pdf]

Supplementary Information

Bearing Fault Diagnosis Method Based on RCMFDE-SPLR and Ocean Predator Algorithm Optimizing Support Vector Machine

Mingxiu Yi [1,2], Chengjiang Zhou [1,2] *, Limiao Yang [1,2], Jintao Yang [1,2] *, Tong Tang[1,2], Yunhua Jia[1,2] * and Xuyi Yuan[3]

1 School of Information Science and Technology, Yunnan Normal University, Kunming 650500, China;

2 The Laboratory of Pattern Recognition and Artificial Intelligence, Kunming 650500, China;

*Corresponding authors.

E-mail addresses: 210007@ynnu.edu.cn (C.Z.)

One Tables and Two Figure Included

Table S1.Analysis of fault recognition rate results under MPA-SVM for RCMFDE-SPLR of different data

method	Data1<br>fs=12000Hz<br>1772r/min<br>motor load 1	Data2<br>fs=12000Hz<br>1730r/min<br>motor load 3
RCMFDE-SPLR<br>MPA-SVM	99.67%	100%

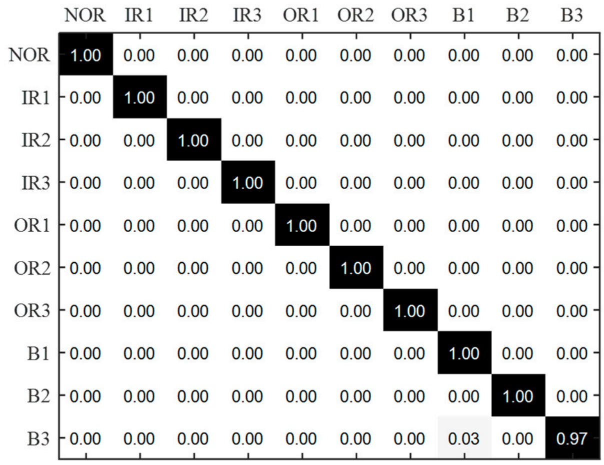

Figure S1. Recognition rate confusion matrix under MPA-SVM for RCMFDE-SPLR of data 1.

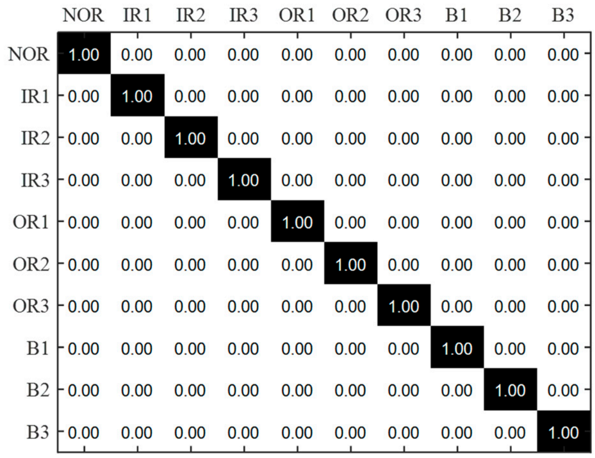

Figure S2. Recognition rate confusion matrix under MPA-SVM for RCMFDE-SPLR of data 2.
